# Abstract—Chairman’s Address

**Published:** 1894-03

**Authors:** A. E. Baldwin

**Affiliations:** Chicago, Ill.


### Abstract—Chairman's Address.

Read in the Section on Dental and Oral Surgery, at the Forty-fourth Annual Meeting of the American Medical Association.

A. E. BALDWIN, M D., D.D.S. CHICAGO, ILL.

The demand for a more thorough education in all departments of medicine stimulates us to action to keep abreast of the times, and we must exercise great wisdom and judgment to always know what new ideas are worthy of fostering and adoption, and what are unworthy of our serious attention. A great danger at present in all walks of life is to fadism, and with us there is danger that in our enthusiastic adoption of some new theory, we do *not* make use of, or realize, the sterling worth of the old, approved and established ideas. We are reminded of this in the now all-absorbing fad of laying the guilt of every ill to micro-organisms, and perhaps in using agents destructive of these, when instead we should use the greatest of all healing and healthful agents alone, and that is cleanliness.

We are further reminded of this in our specialty, when we say there is great danger that in formulating and exemplifying theories of the causes of decay and germ-destroying methods, we may forget that *whatever* the cause of decay, when it is present in the tooth, it should be removed, so that at least in the external walls there be nothing left save that which is healthy, and then by proper adaptation, our work will be as secure from failure as is possible. We do not mean that broad and thorough work and research should be discouraged, but instead, heartily recommended; but what should be discouraged and condemned is the so-called research, which is rather intended to fortify or prove some preconceived idea. The greatest danger in our special field is to narrowness and the lack of patient and earnest search after wisdom in the broader fields. The broader we become as students, the less need will there be of any hampering or stiff boundary lines of rules of ethics, and the more surely the golden rule of "doing as we would be done by" will prevail, and that spontaneously. When we realize how broad the world of wisdom is, we will cease to even imagine great learning in ourselves. We think the attention of the general practitioner should with great emphasis be directed to the necessity of preserving the temporary teeth, especially the temporary molars, for they are not usually replaced by the permanent until the tenth to the twelfth year. The importance of this will be realized, when attention is called to the fact that the child needs well-masticated nutriment much more than does the adult, for their system needs not only material to supply the waste, but also for the rapid growth and development of the whole physical organization. *Again*, if these teeth are neglected, they soon become sensitive, and then the child will not properly masticate the food, but forms the habit of bolting his food, thus introducing the whole train of dyspeptic troubles. *Again*, children's teeth, whether sound or decayed, should only be extracted when the new or permanent tooth to take its place is ready to come in; then only the *one tooth* should be extracted, for in the writer's opinion, one of the most fruitful causes for irregularity in the permanent teeth, is the extraction of more than the one temporary tooth to allow the larger permanent tooth room to come in, forgetting that while coming in apparently with much irregularity oftentimes, they will as a rule regulate themselves by expanding the jaw, before the age of twelve or fourteen years, and this expansion of the jaw is a necessity to the physiologic development of the normal face. And that, recognizing the fact of the natural tendency of the teeth to move forward, or toward the mesial line, the natural sequence of the extraction of a temporary tooth, before the time for the eruption of the corresponding permanent tooth, will be the partial or complete closure of the space, so the permanent tooth when it comes in will have such a restricted space that an irregularity must result.

Another thing is the failure of the general practitioner in not recognizing that, in the great majority of cases, an abscessed tooth may, by proper treatment, be made so that it will give satisfactory service for many years, being almost universally amenable to treatment and filling. So that the field for the judicious extraction of the teeth has been narrowed to a very meager number. And in this connection, the writer expresses the opinion that the persistent, often repeated and long continued treatment of these pulpless teeth is most often needless, and entailing unnecessary expense and trouble to the patient; and that the end may be attained usually in one sitting, *provided*, that the roots can be and are cleaned out and made perfectly dry, and a non-irritable and complete filling placed therein. And if any irritation follows, this can be better treated from without than through the small and often tortuous root-canals. In fact, it is extremely rare that with careful and thorough work, the pericemental irritation, if present at all, will be more than so very slight as to be scarcely noticed.

